# Efficacy and safety of acupuncture for adenomyosis: A protocol for systematic review and meta-analysis

**DOI:** 10.1097/MD.0000000000028080

**Published:** 2021-12-10

**Authors:** Jingru Wang, Tairan Zhai, Xiao Sun, Xinran Du, Xinru Zhang, Xuemeng Shi, Yang Shu, Xiao Yan, Qingchang Xia, Yuxia Ma

**Affiliations:** Shandong University of Traditional Chinese Medicine, Jinan, Shandong, China.

**Keywords:** acupuncture, adenomyosis, dysmenorrhea, hypermenorrhea, infertility

## Abstract

**Background:**

Adenomyosis (AM) is a disease in which the endometrium (including glands and stroma) invades the myometrium and grows. The main clinical symptoms include menorrhagia, dysmenorrhea, chronic pelvic pain, metrorrhagia, and dyspareunia, which will seriously affect the physical and mental health of patients, and most of which occur in women of childbearing age. Acupuncture, as a special external treatment of Traditional Chinese medicine, has shown good effects in the treatment of adenomyosis. At present, there is a lack of systematic review on acupuncture in the treatment of adenomyosis. We conduct this study to evaluate the efficacy and safety of acupuncture in the treatment of adenomyosis.

**Methods:**

We will search Chinese and English databases: Medline, Pubmed, EMBASE, Cochrane library, China National Knowledge Infrastructure (CNKI), Chinese Scientific and Journal Database, Wan Fang database (Wanfang), Chinese Biomedical Literature Database (CBM) to identify articles of randomized clinical trials of acupuncture for adenomyosis. All above electronic databases will be searched from inception to September 30, 2021. RevMan 5.3 software will be used to conduct this systematic review. No language and publication status restrictions will be applied.

**Results:**

The study will prove the efficacy and safety of acupuncture for adenomyosis.

**Conclusion:**

We plan to submit this systematic review to a peer-reviewed journal.

**Trial registration number:**

CRD42021277136.

## 1 Introduction

Adenomyosis (AM) is a disease in which the endometrium (including glands and stroma) invades the myometrium and grows. The main clinical symptoms include abnormal uterine bleeding, dysmenorrhea, and infertility, which will seriously affect the physical and mental health of patients, and most of which occur in women of childbearing age, The incidence of adenomyosis ranges from 7% to 23%.^[1]^ Sex steroid hormone aberrations, inflammation, changes in cell proliferation, and neuroangiogenesis may be the key pathogenic mechanisms.^[2]^ At present, adenomyosis can be divided into diffuse adenomyosis, focal adenomyosis (uterine adenmoysis and uterinecystic adenomyosis) and polypoid adenomyosis (adenomyomatous polyp of the endometrium and atypical polypoid adenomyosis) according to imaging manifestations.^[3,4]^ Pain relief, bleeding reduction, and fertility promotion are the main therapeutic goals of adenomyosis. The treatment of adenomyosis includes medication and minimally invasive/surgical treatment.^[5]^ Medications include non-steroidal anti-inflammatory drugs (NSAID), oral contraceptives, oral hormones, gonadotropin-releasing hormone agonists (GnRH-a), levonorgestrel intrauterine sustained release system (LNG-IUS), mifepristone, progesterone, and oral Chinese medicine. The choice of medication depends on the patient's age, severity of symptoms and fertility requirements, and the combination of individual and standardized drug treatment, long-term efficacy, and adverse reactions should be considered. Traditional minimally invasive/surgical treatments include hysterectomy, uterine artery embolization (UAE), and high intensity focused ultrasound (HIFU). However, hysterectomy is not a good choice for women who want to remain fertile. Although UAE and HIFU treatment can improve patient symptoms, its effects on ovarian function and pregnancy are still uncertain.^[6]^ Radical surgery is only suitable for patients with no fertility requirements and can affect the basin structure and physiological function of the ovary.^[7]^ UAE can effectively relieve dysmenorrhea, reduce menstrual volume, and preserve the function of ovary and uterus. It has remarkable efficacy and is suitable for patients with fertility needs, especially for diffuse adenomyosis.^[8]^ Mifepristone can reduce endometrium pain, bleeding, cell proliferation, and inhibiting inflammation,^[9]^ however, application of mifepristone not adersely affect stimulation, make endometrial estrogen stimulation state for a long time, for more than 3 consecutive months is the for endometrial drug can reduce pain, bleeding of the film inside the uterus, cell proliferation and inhibiting inflammation, no security will need to be further confirmed.^[10]^ The direct antiproliferative effect of GNRH-a can also inhibit the lesion, thereby relieving clinical symptoms.^[11]^ However, menopausal symptoms caused by hypoestrogenemia may occur, such as hot flashes, vaginal dryness, decreased libido, insomnia, depression, etc. Long-term application may lead to bone loss.

As one of the external treatment methods of Traditional Chinese medicine, acupuncture has the functions of dredging meridians, promoting qi and blood circulation, channeling meridians, activating collaterals and relieving pai. Modern studies have found that acupuncture can regulate the endocrine function of female reproduction, it can directly regulate the function of hypothalamic–pituitary–ovarian axis. Stimulating acupoints can release endogenous opioid polypeptides from periaquittal gray matter, and activate opioid receptors in central nervous system to produce labor pains by simulating endogenous antipain substance enkephalin. There are also scholars who think that acupuncture through the adjustment of pelvic nerve plexus, reduce uterine contraction amplitude, relieve uterine muscle spasm, improve pelvic blood circulation, reduce the level of prostagranin, adjust plant nerve function, and have analgesic effect.^[12]^ According to published studies, acupuncture can effectively relieve dysmenorrhea of adenomyosis, reduce menstrual volume, and reduce uterine size. However, there is still a lack of high-quality evidence to support the effectiveness and safety of acupuncture on adenimyosis. Based on this, we will systematically compare the efficacy and safety of acupuncture in the treatment of adenomyosis, there by paving the way for the future treatment of adenomyosis.

## 2 Methods and analysis

### 2.1 Study registration

This systematic review protocol has been registered in the PROSPERO network (No. CRD42021277136). And it will follow recommendations outlined in The Cochrane Handbook of Systematic Review of Interventions.

### 2.2 Criteria for including studies

#### 2.2.1 Types of studies

Randomized controlled trials of acupuncture in the treatment of adenomyosis will be comprehensively searched in both Chinese and English databases. In addition, unpublished documents will be searched manually. Non-randomized controlled and observational studies will be excluded. Animal studies, reviews and case reports will be excluded.

#### 2.2.2 Types of participants

Patients with adenomyosis on gynecological ultrasound, aged between 18 and 50 years old. Not restricted by race and nationality.

#### 2.2.3 Types of interventions

Patients in the treatment group should be treated with acupuncture alone. Patients in the control group will receive other treatment without acupuncture.

#### 2.2.4 Types of outcome measures

##### 2.2.4.1 Primary outcomes

The degree of dysmenorrhea relief, changes in menstrual volume and uterine volume were the primary outcomes.

##### 2.2.4.2 Secondary outcomes

1. Abdominal pain score (visual analogue Scale, VAS score)
2. Dysmenorrhea accompanies symptoms
3. The value of serum carbohydrate antigen CA125
4. Frequency of changing sanitary napkins
5. Blood covers the area of sanitary napkins in 1 h

### 2.3 Search strategy

We will search the following databases: Medline, Pubmed, EMBASE, Cochrane library, China National Knowledge Infrastructure (CNKI), Chinese Scientific and Journal Database, Wan Fang database (Wanfang), Chinese Biomedical Literature Database from inception to September 30, 2021. According to different databases, we combine keywords and free words for comprehensive search. The search strategy in Medline is as follows (Table 1).

**Table 1:** Medline search strategy.

Number	Search items
#1	MeSH major topic:adenomyosis
#2	MeSH major topic:endometriosis
#3	MeSH major topic: dysmenorrhea
#4	MeSH major topic: dysmenorrhea relief,
#5	MeSH major topic:infertility
#6	MeSH major topic:acupuncture
#7	MeSH major topic:Women of childbearing age
#8	MeSH major topic:women
#9	MeSH major topic:menorrhagia
#10	MeSH major topic:serum carbohydrate antigen CA125
#11	MeSH major topic:serum CA125
#12	MeSH major topic:uterus
#13	MeSH major topic:uterine volume
#14	#1 or #2
#15	#3 or #4
#16	#7 or #8
#17	#10 or #11
#18	#12 or #13
#19	#5 and #6 and #9 and #14 and #15 and #16

### 2.4 Data collection and analysis

#### 2.4.1 Selection of studies

**Figure 1:**
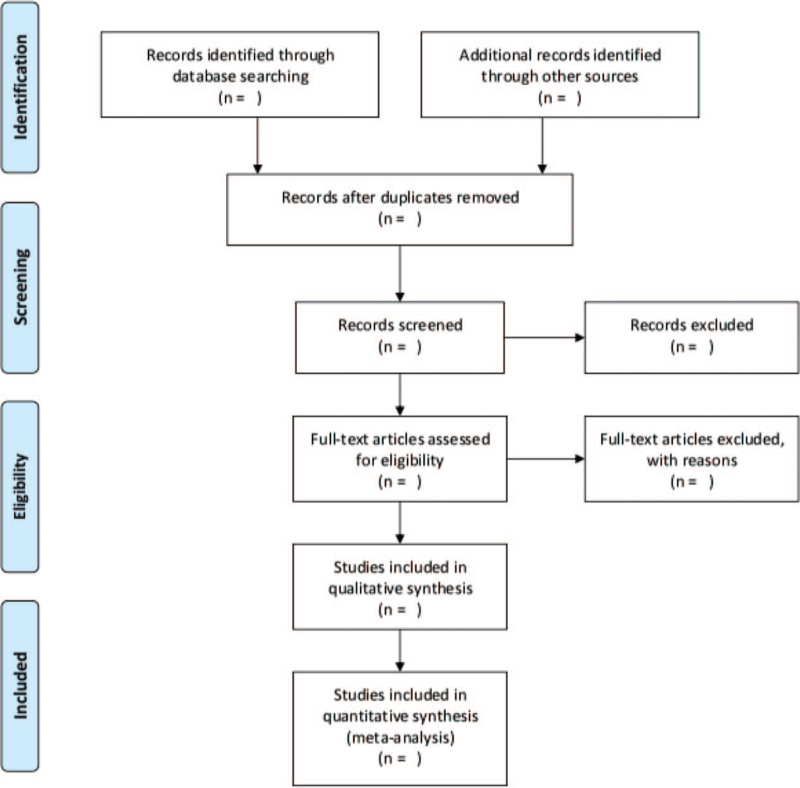
The PRISMA flow diagram.

We will deal with the included literature in the following way and the specific operation is as follows: First, according to the selected topic, the retrieved literature is imported into note Express 3.0 in the document manager in the correct retrieval way, and remove the duplicate published article; then, by reading the title and abstract one by one, the article irrelevant to this research will be weeded; then, the remaining articles will be downloaded in sequence and the full text will be read; finally, according to the inclusion and exclusion criteria required in this paper, inductive statistics, and form the final paper. The two researchers will strictly follow the above procedures to collect and delete the literature. If there is any disagreement, they will negotiate with the third evaluator to finalize the results. The included article process is shown in Figure 1.

#### 2.4.2 Data extraction and management

Two researchers from the database for data extraction and data management, including the title, year of publication, the first author surname, general information, research design, the research cycle, sample size, test group and control group intervention methods, treatment time, the result, adverse events, if two researchers any inconsistencies in the process, will make our final decision by team members to discuss.

#### 2.4.3 Dealing with missing data

If the data is lost in the literature, we will do our best to contact the corresponding author for more detailed information. If the missing data is not available, we will exclude these articles and integrate the rest of the research.

#### 2.4.4 Assessment of risk of bias

In this work, Cochrane Handbook for systematic reviews of interventions Version 6 will be used to assess a broad category of biases. Two researchers will determine the bias based on the following items: random sequence generation, allocation concealment, blinding of the participants and personnel, blinding of the outcome assessments, incomplete outcome data, selective reporting, and other sources of bias. The studies will be evaluated as “Low risk,” “High risk,” or “Unclear risk.” Inconsistencies will be resolved by discussion with other reviewers.

#### 2.4.5 Measures of treatment effect

All valid data will be transmitted to Stata 15 for analysis and synthesis. For continuous results, the data will be expressed as mean difference (MD) or standard mean difference (SMD) with 95% confidence interval. When there are dichotomous data, a hazard ratio with a 95% confidence interval will be used. When binary data exists, the RR format will be changed for analysis.

#### 2.4.6 Assessment of quality of evidence

It is recommended that the Assessment, Development, and Evaluation Score (GRADE) will be used to evaluate the results of this system review. In the GRADE system, the quality of evidence will be defined as high, moderate, low, or very low.

#### 2.4.7 Assessment of reporting bias

We will use the funnel plot and Egger test to assess publication bias if >10 articles are included.

#### 2.4.8 Assessment of heterogeneity

The heterogeneity between the results included in the study will be calculated by Cochrane *X*^2^ and *I*^2^ tests. If *P* ≥ .05 and *I*^2^ < 50%, the statistical heterogeneity between these studies can be ignored. If *P* < 0.05 and *I*^2^ > 50%, it is considered that there is great heterogeneity between these studies.

#### 2.4.9 Data synthesis

We will take advantage of Review manager software (RevMan) V.5.3 for data analysis and synthesis. If there is no statistical heterogeneity between the results, a fixed effect model will be used. If there is a statistical heterogeneity between the results, a random effect model will be used. If there is a significant clinical heterogeneity, subgroup analysis, or sensitivity analysis is performed.

#### 2.4.10 Sensitivity analysis

We will carry out sensitivity analysis to investigate the robustness of the study conclusions. The principal decision nodes include method quality, sample size, and the impact of missing data. The meta-analysis will be repeated, and low-quality studies will be excluded. Therefore, the impact of low-quality studies on the overall results will be evaluated.

#### 2.4.11 Subgroup analysis

Where possible, we will conduct subgroup analysis based on different interventions, age, gender, duration of treatment, and outcome measures.

#### 2.4.12 Ethics and dissemination

This work does not require ethical approval, because there is no data related to individual patient data in our study. The result will only be released in publications of peer reviews.

## 3 Discussion

Adenomyosis is characterized by progressive enlargement of the uterus, increased serum CA125 level, dysmenorrhea, menorrhagia and infertility, which has a serious impact on women's lives. Although surgery and drug therapy can achieve a certain effect, but with varying degrees of side effects, patients will also bear the high cost of surgery and drug costs. As one of the external treatment methods of Traditional Chinese medicine, acupuncture has the characteristics of simple and simple verification. It can not only prevent the occurrence of diseases, but also be used as an auxiliary treatment after the occurrence of diseases. Therefore, we will conduct a systematic review and meta-analysis of randomized controlled trials to verify that acupuncture can treat adenomyosis or relieve the clinical symptoms of adenomyosis. We hope this review will provide more convincing evidence for clinicians to treat these conditions and help them make decisions.

## Acknowledgments

This study was supported in part by the National Natural Science Foundation of China (No. 81774402).

## Author contributions

**Data curation:** Jingru Wang, Tairan Zhai.

**Formal analysis:** Xiao Sun, Xinran Du.

**Methodology:** Jingru Wang, Tairan Zhai, Xinru Zhang, Xuemeng Shi.

**Project administration:** Yuxia Ma.

**Resources:** Jingru Wang, Tairan Zhai, Xinru Zhang, Yang Shu, Xiao Yan.

**Software:** Jingru Wang, Qingchang Xia.

**Visualization:** Qingchang Xia.

**Writing – original draft:** Jingru Wang, Yuxia Ma.

**Writing – review & editing:** Yuxia Ma.
